# Mitral valve repair with the use of the “Memo 3D ReChord” ring

**DOI:** 10.1186/s13019-023-02200-w

**Published:** 2023-04-17

**Authors:** Nikolaos G. Baikoussis, Nikolaos Koumallos, Κonstantina Aggeli

**Affiliations:** 1 Cardiac Surgery Department, Ippokrateio General Hospital of Athens, 114 Vasilissis Sofias Avenue, Athens, 11527 Greece; 2 1st Department of Cardiology, Ippokrateio General Hospital of Athens. Athens University, School of Medicine, Athens, Greece

**Keywords:** Mitral valve repair, Memo 3D ReChord, Mitral annuloplasty, Neo-chords in mitral valve, Chordae rupture, Mitral valve regurgitation

## Abstract

**Background:**

From a variety of ring types, semirigid ring is more preferred for mitral annuloplasty during mitral valve repair particularly in patients whose native mitral saddle shape annulus is well maintained. During mitral annuloplasty artificial chord implantation with the appropriate neochord length is surgically challenging. We present our experience of using the Memo 3D ReChord, a semirigid ring with additional chordal guiding system for mitral valve repair.

**Patients and methods:**

From September 2018 to February 2020, we successfully treated ten patients with severe (4+/4+) degenerative mitral valve regurgitation due to posterior leaflet prolapse with chordal rupture with the implantation Memo 3D ReChord and neo-chords.

**Results:**

We implanted from one to three neo-chords and always a ring in our patients. None of the patients had any residual mitral valve regurgitation at the end of the repair and on their discharge evaluated through transesophageal and transthoracic echocardiography respectively. There was no mortality at 30-days or on midterm follow-up. During the 3-month follow-up no regurgitation was noticed either. We included in our study only the patients successfully treated. We also used it in two patients, who underwent valve replacement during the same operation due to mild to moderate mitral valve regurgitation.

**Conclusions:**

This, in our knowledge, is the first Greek series of the implantation of the Memo 3D Rechord. The initial excellent results give us the enthusiasm to continue while long-term results and the durability of this technique are necessary to establish this semirigid annuloplastic ring in our every-day practice.

## Introduction

Mitral regurgitation (MR) is the second-most frequent valvural heart disease (VHD) in Europe, with degenerative aetiology particularly due to fibroelastic deficiency and Barlow disease being the most common in Western countries. According to the 2021 ESC/EACTS Guidelines for the management of valvular heart disease, surgery is highly recommended, Class I, in patients with symptomatic severe degenerative MR and acceptable surgical risk, with mitral valve repair (MVr) being the surgical intervention of first choice [1]. Alain Carpentier in Paris France intuited most of the techniques for MVr started his study in 1970, introducing the remodeling of the annulus with ring annuloplasty and the well-known triangular or quadrangular resection of leaflet tissue for restoring leaflet coaptation [2].

There are many kinds of rings used for commonly mitral annuloplasty during MVr. Some rings are complete, saddle-shaped, incomplete or semirigid. The more suitable the ring is, the better the long-term durability of the MVr will be [3–6]. Since there is no clear indication so far, it is on the surgeon’s preference which one to choose. The use of saddle-shaped annuloplastic ring has been suggested for diminishing mitral annular and leaflet strain, and improving leaflet coaptation geometry [3, 6–9], while for others remains controversial [10]. No indication has been reported in guidelines.

During MVr, artificial chord implantation has become one of the most applied techniques. While resection of flail or prolapsing leaflets were applied in the past, implantation of artificial neo-chords, has been introduced with results equivalent to those of resection [11–13]. Choosing the appropriate length of artificial chords has always been a challenging step during the annuloplasty. Intraoperative saline testing, preoperative transesophageal echocardiography (TEE) and intraoperative caliper measurement, the “loop technique”, have been proposed to determine the distance between the papillary-muscle head and the free-margin of the leaflet [11, 13, 14].

**Fig. 1:**
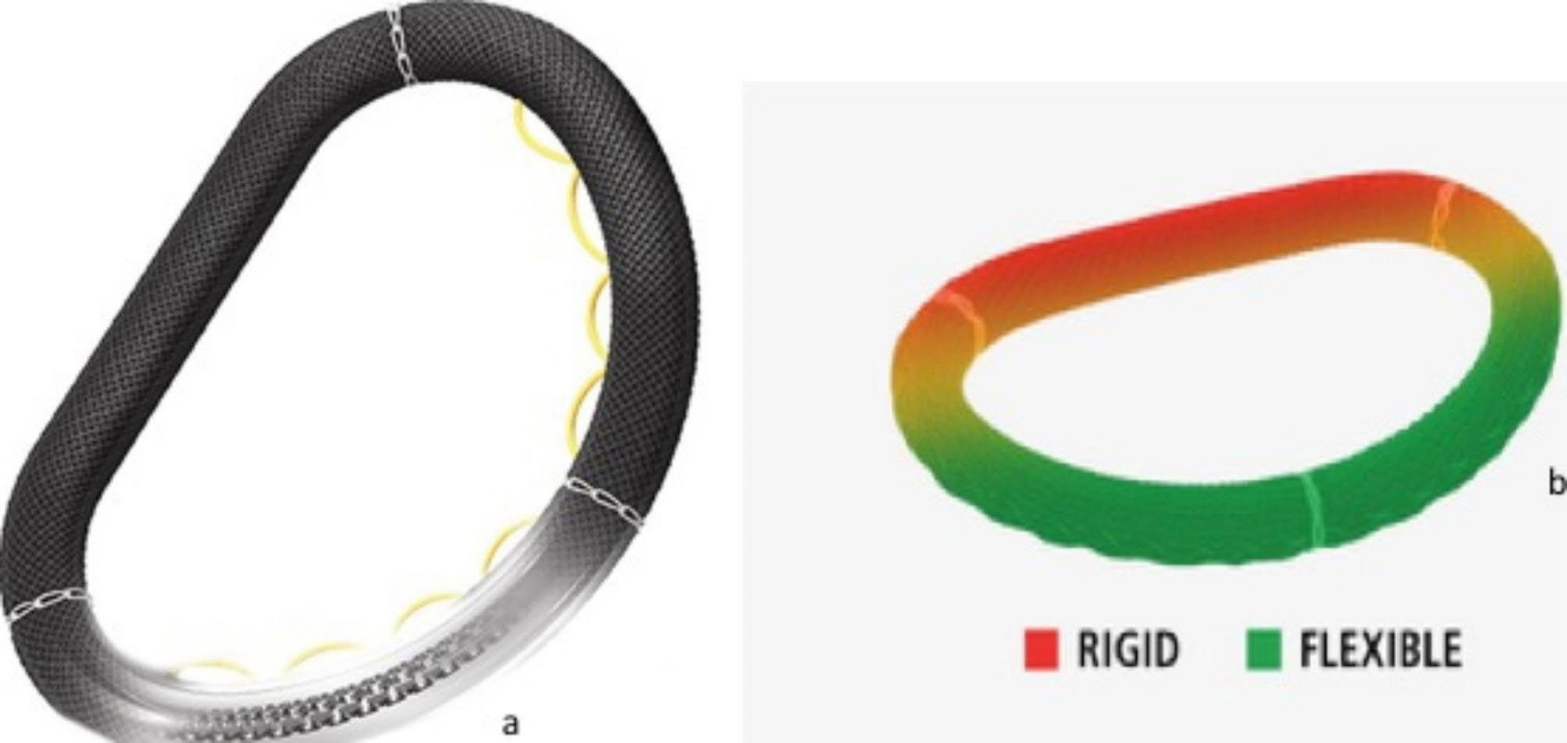
**1a:** The Memo 3D ReChord annuloplasty ring with its temporary chordal guiding system (yellow loops). **1b:** The above described ring is semi-rigid; it means that the anterior part (red) is rigid and the posterior (green) became progressively flexible mimicking the native annulus of the mitral valve

The semirigid complete ring Memo 3D ReChord (LivaNova, London, United Kingdom) (Fig. 1) is a semi-rigid ring with a chordal guiding system, which has the aim to simplify the implantation of the artificial polytetrafluoroethylene (PTFE) neo-chordae leading to accurate and successful implantation results, without the need of specialized chordal measurement [3, 11].

## Patients and methods

From September 2018 to February 2020, we implanted the Memo 3D ReChord rings in 10 selected patients aged 46–72 years who had severe (> 3 + or 4+/4+) degenerative MR due to posterior leaflet prolapse. In patients with both posterior and anterior leaflet prolapse we proceeded with valve replacement. The objective of this study was to summarize our initial experience with Memo 3D ReChord rings for MVr. Inclusion criteria: We included patients with MR due to degeneration, and rupture of the chordae of the posterior leaflet of the mitral valve needed new chords implantation. Exclusion criteria: We excluded patients with mitral valve stenosis, endocarditis, emergent cases, patients with secondary mitral valve insufficiency, ischaemic mitral valve regurgitation and severe prolapse of both posterior and anterior leaflets. No age, sex or nation limitation were considerate. Preoperatively transthoracic (TTE) and transesophageal (TEE) echocardiography were performed in every patient in order to become candidate for mitral valve surgery [1, 15].

In operating room, just before sternotomy we also performed a 3D TEE to obtain a better “surgical view” of the anatomical structure responsible for the MR. In this way it was possible to define exactly the corrections needed, as for example; gap between P1-P2, chordae rupture, excessive valve tissue in any leaflet, other than the evaluation of tricuspid valve. We performed 3D TEE before and after mitral valve repair in real haemodynamic conditions with the heart beating. Once medial sternotomy was performed, we fully heparinazed the patient and bicaval cannulation was done. We used antegrade Custodiol as cardioplegic solution at 15–20 ml/kg body weight and non-more than 2 litters as a single dose providing a long period of myocardial protection. In this way we achieved excellent myocardial protection for 2 h without disturbances of the surgical field every 20 min. In case more cross-clamp time was required to supplement corrections or replacement of the valve due to unsuccessful repair, myocardium was already well protected. All the procedures were performed in mild hypothermia (32-34^0^ C). The corrections been made via left or right atriotomy depended on the tricuspid valve pathology or the heart anatomy of any patient. The mitral valve was checked with water test and nerve hook in order to evaluate the pathology and the correction strategy.

**Fig. 2:**
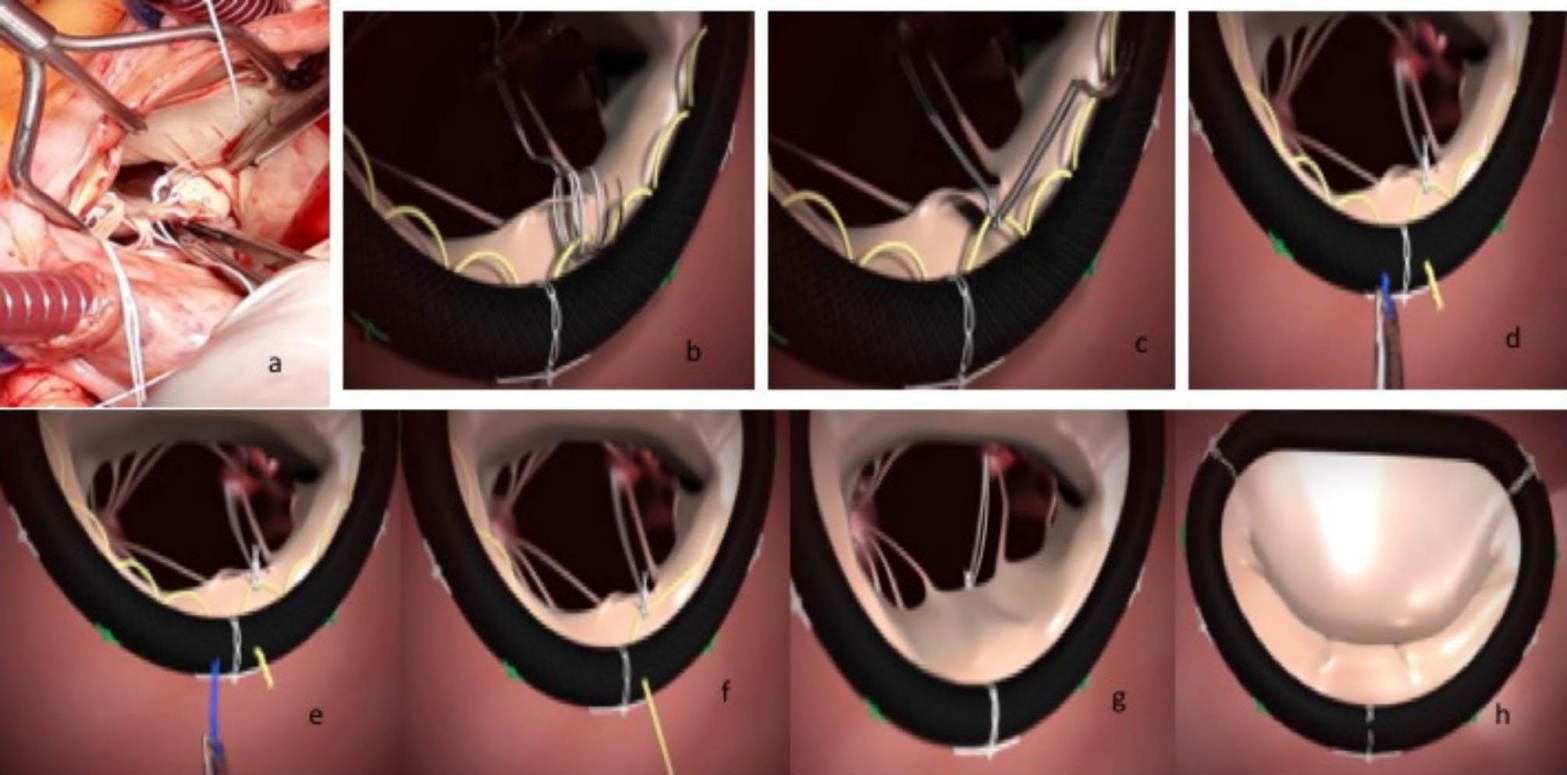
**2a:** Implantation of the artificial neo-chordae CV4 at the top of the corresponding papillary muscle **2b,c:** The ring is sutured on the annulus. The two needles of the double armed neo-chordae were passed through the posterior leaflet of the mitral valve and then through the yellow loops of the guiding system of the ring **2d:** The neo-chordae sutures are tied and the needles are cut just above the nodes **2e,f:** The temporary chordal guiding system (blue suture and yellow loops) is removed. The blue suture is pulled out first. Then, is pulled out the yellow loop) **2 g:** The posterior leaflet is free in order to obtain its final normal position **2 h:** The final result at the end of the repair with the “smile” of the competent mitral valve

The semi rigid Memo 3DReChord annuloplasty ring (Fig. 1) was used, with its innovative chordal guiding system that enhances procedural control particularly when performing artificial chordae replacement is needed. With this device, the posterior annulus was used as a reference point for the height of the artificial neo-chorda. This technique relies on the principle of basal marginal chordae equivalence, where the height of a marginal (primary) chorda is always equal to that of the corresponding basal (tertiary) chorda [16]. We started with the implantation of the neo-chordae at the top of the corresponding papillary muscle where the PTFE neo-chordae Gore-Tex sutures (WL Gore & Associates, Flagstaff, AZ, USA) were attached (Fig. 2a). We measured the annulus, and we implanted the proper ring with great attention to non-downsizing the annulus, since the latter may be the reason of systolic anterior motion, especially if a kind of anterior leaflet prolapse is present, thus obstruction of the outflow of the left ventricle and consequently presence of residual MR. Then, the two needles were passed through the mitral leaflet and the yellow loops of the guiding system of the ring (Fig. 2b and c). The free margin (coaptation point-line) of the posterior leaflet was brought to the posterior annulus (Fig. 2d), the PTFE (CV4 or CV5) sutures was tied underneath the mitral leaflet on the ventricular side (Fig. 2d), and the temporary yellow loops of the ring were removed (Fig. 2e and f). The length of the neo-chordae obtained with this system exactly matched the plane of the native annulus at the coaptation line (Fig. 2g). This is the description of the innovative ring and the technique for its implantation. At the end of the repair, we should see the “smile” of a competent mitral valve (Fig. 2h). We also tested the competence of the valve with water irrigation, and we carefully inspected the valve.

In our series, 4 patients had only P2 prolapse, 3 had P1 and P2 prolapse and in other 3 had P2 and P3. Three patients required additional corrections like sutures in order to close a huge gap between P1-P2 or P2-P3. We implanted two Memo 3D ReChord sized 30 mm, four Memo 3D ReChord sized 32 mm and four Memo 3D ReChord sized 34 mm. Our patients required from one to three neo-chordae. In 3 patients we corrected the valve with only one chorda, in 3 with two chordae and in 4 patients with 3 neo-chordae other than the ring always implanted. Concomitant surgical interventions were required in 3 out of 10 patients, who also underwent replacement of the tricuspid valve with a ring implantation. Closure of the left atrial appendage was performed in two patients with atrial fibrillation with the Gillinov-Kosgrove clip (AtriCure Inc, Westchester, OH, USA). No coronary artery bypass grafting, and no intra-aortic balloon pumping were needed. We started our program with the correction of the posterior leaflet prolapse considered simple as pathology and as surgical procedure since the correction of the anterior leaflet is considered more complex [2]. We implanted 2 rings in patients with posterior leaflet prolapse but the TEE at the end of the repair revealed moderate regurgitation and we replaced the valve during the same operation. The mechanism of residual MR was overcorrection of the posterior valve due to tight knots and systolic anterior motion due to downsizing of the annulus. The second case, we notice a destroy of the leaflet during manipulation. They all were operated on by the same surgeons of this series (N.G.B and NK). If sinus rhythm was established, anticoagulation with warfarin was indicated for three months only. The day of dischargement (4th or 5th postoperative day) a TTE was performed in all patients. Nobody had any regurgitation. In 1-mounth and 6-month follow up the mitral valve was competent without any insufficiency.

## Results

We successfully implanted the Memo 3D ReChord in 10 patients. Cross clamp time was obviously longer than the usual ishaemic time for mitral valve replacement (94 vs. 58 min respectively). This is the main reason for what we used Custodiol, a long-acting intracellular crystalloid cardioplegia, as cardioplegic solution during MVr. No residual MR was observed in any patient. In two patients with moderate regurgitation at the end of repair, we proceeded with valve replacement immediately and we excluded them from our series. No patient needed postoperative re-exploration for bleeding or required permanent pacemaker implantation. All patients were discharged on mild diuretic drugs, angiotensin-converting enzyme inhibitors and anticoagulation treatment (warfarin) for 3 months. Those being in sinus rhythm received aspirin 100 mg daily. Warfarin life-long was administered only to those with chronic atrial fibrillation. No mortality occurred at 30-days or on 3-month follow-up. Pre-operative, intra-operative TEE and postoperative (on discharge day) 3D TTE were complete in all cases, with subsequent 3-month TTE follow-up (some 6-month follow up). No patient had any symptoms or other valvular pathology.

## Discussion

This is an institutional report, and our aim is to present our initial experience in MVr with the use of the Memo 3D ReChord ring. Successful implantation with no residual MR and length determination of neochords using the loops on the posterior aspect of the annuloplasty ring as the reference point, were feasible in all patients. To the best of our knowledge this is the first Greek report and first Greek series in MVr with this ring.

The semi-rigid device is an upgrade to the existing Memo 3D ring. Semirigid means that the anterior portion of the ring is rigid and the posterior progressively flexible mimicking the native annulus of the mitral valve. This is a special characteristic of this ring in order to be as possible physiologic as it can. The ring is covered with Sorin’s unique Carbofilm coating that, according to the company, improves the bio and hemo-compatibility of the ring. The exclusive alloy core cell design is a laser-cut one-piece structure that allows the physiological three-dimensional motion of the mitral annulus with a natural anterior/posterior to lateral/lateral relationship maximizing the blood flow. The oval silicone sheath provides easy suturability with conformable needle penetration. The bio and hemo-compatible properties of the unique Carbofilm coating allows complete endothelialization, prevents inflammatory reaction and scar tissue formation. The additional chordal guiding system of the ring provides that the length of the neo-chordae obtained matches exactly the plane of the native annulus at the coaptation line leading to excellent results of the artificial chordae replacement.

In our series, we implanted only for posterior leaflet prolapse. We freely recommend the use of this ring with some precautions especially during the learning curve; the use of Custodiol as long-acting intracellular crystalloid cardioplegia for better myocardiac protection, the preoperative TEE in the operating theater in order to evaluate perfectly the valve in “surgical view”, the TEE at the end of the repair in real time conditions, meaning real and ideal hemodynamic profile. If moderate regurgitation is found, a second attend to repair is recommended if feasible or valve replacement without prolong much more the ischaemic time. We should always keep in mind that we are treating patients with severe valvulapathy and possible low or moderate ejection fraction of the left ventricle. For this reason, myocardial protection is crucial during mitral valve surgery. What is more, a number of MVr per year is mandatory in order to maintain a certain surgical skill in this procedure. In our institution, we extremely rarely perform triangular or quadrangular resection but, if necessary, we perform only some resection of a ruptured chordae or any excessive tissue. We apply the dogma “respect rather than resect” and of course “if resect, resect with respect” according P. Perier techniques [17].

According to the literature a semirigid ring provides better results in patients with severe degenerative MR who require annuloplasty during the MVr [3, 18, 19]. What is more, the Memo 3DReChord ring has the advantage to maintain annular motion and allow annulus remodeling, reflected in the restored annular saddle shape during the systolic phase, as well as the normal annular physiological relationship (vertical/transverse 1⁄4 3:4) [3, 18, 20–22]. Santarpino et al. reported a case of Sorin Memo 3D ring implantation, in which mitral annular flexibility was still preserved at 5 years of follow-up [23]. With this in mind, the Memo 3D ring appears to be superior to the C-shaped incomplete band or the flexible full ring [3, 18–20].

The Memo 3DReChord can be used for both posterior and anterior leaflet prolapse. The anterior leaflet has a narrower attachment to the mitral annulus but also a much broader occlusive surface area when compared to the posterior mitral leaflet [10, 18]. For this reason, the reconstruction of the anterior mitral leaflet is considered more challenging and thus the precise measurement of the artificial neo-chorda such the one offered by the innovative chordal guiding system of the Memo 3DReChord ring, is of critical importance for the excellence of the surgical procedure [24, 25]. Glauber et al. reported that with the Memo 3DReChord device, as the posterior annulus is used as the reference point for length determination of the neochords, the length of the neochords matches exactly the coaptation point, independently if used for anterior or posterior mitral leaflet leading to better procedural control and outcome of the artificial chordae replacement during minimaly invasive MVr [25].

MVr is a challenging procedure which requires experienced surgical team and medical centers. Even though advances in annuloplasty technology have been made, the surgical technique may also lead to an unwanted outcome. It is for this reason that we must stress the necessity of correct training in order to minimize this factor [4, 26]. In another series using the Memo 3DReChord, Prinzing et al. noticed some technical considerations for the successful use of the ring. They suggested that examination of the intraventricular course of the neo-chords should happen before the neo-chord is tied to the yellow loops of the ring device in order to avoid a too long implanted neo-chord. Attention not to overextend the neo-chords should also be given, especially when the papillary muscle head is pulled toward the mitral valve plane. Additional tension of the neo-chordae may lead to overcorrection and restriction of the diseased segment. A mark should be used to the exact area of chorda implantation during water testing to avoid any misplace, since repositioning is not an option after [11].

The Memo 3DReChord device requires a learning period so that the surgeon obtain surgery experience with high repair rates, low operative mortality, and a record of durable results. Our aim as a center, is to perform minimal invasive mitral valve repair through right mini and video-thoracoscopic access in order to minimize the local and systematic complications [27]. In our department we already performed transapical chordae implantation for MVr [28] and other innovative techniques.

In conclusion, through this study we would like to underline some important issues; the feasibility of MVr especially of the posterior leaflet prolapse after good preoperative evaluation, the use of Custodiol for myocardial protection in MVr and the importance of the TEE at the beginning and at the end of repair in order to have the confirmation of a perfect result and a competent mitral valve.

Our experience showed that this annuloplasty device has an excellent mitral valve competency and its artificial chordae guiding system offers correct determination of length, resulting to successfully early clinical outcomes from our series. We express our enthusiasm in its results and hemodynamic behavior while waiting for larger series using the Memo 3DReChord ring to come.

## Data Availability

available in the institute.

## Abbreviations

MR: Mitral regurgitation
MVr: Mitral valve repair
PTFE: Polytetrafluoroethylene
TEE: Transesophageal echocardiography
TTE: Transthoracic echocardiography
